# The association of financial resources and loneliness among older adults during a state of emergency

**DOI:** 10.1371/journal.pone.0314042

**Published:** 2025-01-09

**Authors:** Madeleine Drost, Cäzilia Loibl, Anastasia Snyder, Michael Betz

**Affiliations:** 1 The John Glenn College of Public Affairs, The Ohio State University, Columbus, Ohio, United States of America; 2 Department of Human Sciences, College of Education and Human Ecology, The Ohio State University, Columbus, Ohio, United States of America; Sunway University, MALAYSIA

## Abstract

This study focuses on the initial wave of the COVID-19 pandemic in Spring 2020 in the United States to assess how liquidity constraints were related to loneliness among older adults. Data are from the COVID Impact Survey, which was used to collect data in April, May and June 2020 across the U.S. (n = 5,664). We use means comparison tests and linear regressions and find that emergency savings, rather than household income, predict loneliness among older adults during the initial COVID-19 wave. Emergency savings, especially enough to avoid using credit cards, was most predictive of older adult loneliness levels. Income and access to emergency savings did not influence the relationship between actions taken and personal plans changed as a result of COVID-19. Easing lockdown restrictions was unrelated to the relationship between loneliness and liquidity constraints, actions taken and personal plans changed due to the COVID-19 pandemic. Findings suggest that, in the early months of the COVID-19 pandemic, loneliness associated with the actions taken to avoid COVID-19 and personal plans changed was experienced across all socio-economic groups of older adults in this sample in similar ways, regardless of income levels and wealth. In addition, a better understanding of loneliness in older age during the COVID-19 pandemic may require a fuller analysis of households’ financial situation beyond income, and points to the central role of credit card debt for loneliness in older age.

## 1. Purpose

The purpose of this study is to understand how older adults experienced loneliness during the initial waves of the COVID-19 pandemic and how these experiences were mitigated or enhanced by financial resources.

## 2. Background

About 56% of older adults report experiencing loneliness in the United States during the initial wave of the COVID-19 pandemic [1]. Loneliness, often defined as the feeling of being isolated, has been shown to be higher among older adults than other population groups [2]. Evidence suggests that loneliness is associated with significant health risks, including higher levels of depression, anxiety, thoughts of suicide, functional decline, and shorter life expectancy [2]. Consequently, understanding the determinants of loneliness in older age is an important area of research.

During the COVID-19 pandemic—especially during its initial wave—42 states and territories issued mandatory stay-at-home orders and stressed social distancing for older adults due to their high vulnerability to severe illness and death from COVID-19 [3]. As a result, older adults took a number of actions that likely impacted their experience of loneliness. For example, they reduced their contacts with friends and family, limited their social life [4–6], avoided health care visits [7], and exited the labor force [8]. In this study, we examine the financial situation of older adults to investigate whether and to what extent liquidity constraints were related to reporting loneliness. We focus on the initial wave of COVID-19, which is typically defined from March to August 2020 [9], and consider both household income and lack of emergency savings to measure liquidity constraints. We take into account the extent to which older adults took actions to avoid a COVID-19 infection and changed personal plans due to the COVID-19 pandemic. We also test whether easing lockdown restrictions was associated with loneliness.

The specific research questions are: (1) Did household income and emergency savings predict loneliness among older adults during the initial COVID-19 wave in Spring 2020? (2) Which type of liquidity constraints were most predictive of older adults’ loneliness during the initial COVID-19 wave? (3) Did the relationship between personal actions taken to avoid a COVID-19 infection and loneliness differ by household income and emergency savings? (4) Did easing lockdown restrictions after the initial COVID-19 wave affect the relationship between loneliness and liquidity constraints, actions taken, and having changed personal plans due to the COVID-19 pandemic?

Data for the analysis come from a national three-wave repeated cross-sectional survey in the U.S. The results indicate that lack of emergency savings, especially the need to use credit cards to meet unexpected expenses, predicted loneliness among older adults during the initial COVID-19 wave. Actions taken by older adults to avoid a COVID-19 infection and changing personal plans due to avoid COVID-19 were associated with loneliness, regardless of household income and emergency savings, that persisted after lockdown restrictions were eased. The results highlight the importance of liquidity constraints when studying loneliness in older adults, especially during stressful events like the COVID-19 pandemic.

## 3. Literature review

For our literature review, we followed the Preferred Reporting Items for Systematic Reviews and Meta-Analyses (PRISMA) approach [10]. We used Elsevier Scopus database, which includes the relevant peer-reviewed journals for our topic [11]. We conducted our literature search over several months to ensure newly published studies are included and include studies published before March 7, 2024. Our inclusion criteria were that studies examine financial situations as a predictor of loneliness, with loneliness as the outcome measure; studies with data collected in the United States and similarly developed countries; and studies that examined data from the initial COVID-19 wave during Spring 2020; and article language is English; studies were published from 2020 to present.

Exclusion criteria included studies solely using pre-COVID-19 data; data collected in less developed countries; studies based on younger adults; studies which do not use loneliness as an outcome measure but as a predictor; and commentary-style studies that are not based on original data. The keyword strings “covid AND older AND adult* AND lonel* AND financ*” resulted in 52 studies and “covid AND older AND adult* AND lonel* AND socioeconom*” resulted in 42 studies in the Elsevier Scopus database. The article abstracts were read and after inclusion criteria were applied, 26 articles were downloaded and reviewed. A total of 14 articles met all inclusion criteria and were included in the literature review, see S1 Table in S1 File for a detailed list.

Five of the 14 studies identified in the literature review were based on U.S. data and collected data during the first 2020 COVID-19 year. Four of these studies used national samples [12–14], and one used a subnational sample [15]. Generally, the studies found a link between liquidity constraints and loneliness during COVID-19. Data of 3,961 older adults from the National Health and Aging Trends Study showed that financial difficulty and income decline during COVID-19 were associated with higher risk of loneliness [13].

The COVID-19 Coping Study of 6,938 adults age 55 and older focused on employment status and found that unemployed status at the start of the pandemic and pandemic-era under- and unemployment was associated with higher risk of loneliness [12]. Data collected through crowdsourcing survey platforms of 501 adults age 60 and older documented that financial instability (“having just enough to get along” and “can’t make ends meet”) was associated with higher risk of loneliness [14].

Data from the nationally representative Understanding America Study COVID-19 Survey from April and May 2020 of 3,253 adults 50 years or older showed that the risk of loneliness was higher for older adults in the highest, over $100,000 household income group, but only if the personal contact with other household members was avoided due to COVID-19, as indicated in interaction terms [6]. Data collected mostly in Michigan (83% of sample) of 701 adults age 50 and older confirmed that “financial problems created by pandemic-related changes” were linked to higher risk of loneliness [15]. Taken together, these studies indicate a relationship between a higher risk of loneliness and financial problems and income decline due to under- and unemployment during the initial COVID-19 wave.

Eight studies used data from Canada, Europe, or Japan and confirmed the link between measures of liquidity constraints and risk of loneliness during the initial COVID-19 wave. The Caregiving, Aging, and Financial Experiences study in Canada collected data at two points from 4,010 and 2,420 adults aged 65 and older and showed that financial strain (“past 3 months had trouble paying the bills; not have enough money to buy household needs; finances not worked out by the end of the month”) was associated with greater risk of loneliness. Additional analyses showed that greater feelings of mattering and self-esteem attenuated the main association [16]. Eurofound COVID-19 survey data of 31,757 adults aged 50 years and older in 27 countries in Europe provided strong evidence of a direct link between change in finances and difficulties paying for basic necessities with a three-item psychological distress scale of which “felt lonely” was one item (also included: “felt tense”, “felt depressed”; alpha = 0.806) [17]. No results were provided for the loneliness measure but itself, but the robust study design points to a likely association [17].

A U.K. study used four national data panel studies ranging from 763 to 4,440 adults age 50 and older to examine loneliness over two time points prior to and during the initial COVID-19 wave [18]. Results indicated groups with higher financial vulnerability, including women, renters, unemployed, older adults with health conditions, or greater financial stress, report higher risk of loneliness consistently before and during the pandemic with slight increases during the pandemic. A second U.K. study focused solely on data of 5,146 adults age 50 and older in the English Longitudinal Study of Ageing before the COVID-19 pandemic (2018 and 2019) and at two data points in 2020 [19]. Findings showed that increases in loneliness during COVID-19 were smaller for adults in the lowest wealth group, compared to the highest wealth group, noting that the lower wealth groups reported overall higher levels of loneliness before the pandemic and during 2020 [19].

Data of the Elderly Care Survey 2019 and 2020 in Sweden surveyed 205,529 adults aged 65 and older in nursing homes or home care. Interestingly, results indicate a lower risk of loneliness among groups with lower disposable incomes compared to groups with higher disposable incomes in 2019 pre-pandemic [20]. During the pandemic, in 2020, both lower and higher income groups reported similar levels of loneliness. The study’s focus on diverse segments of older adults in Sweden concludes “surprisingly low levels of loneliness in some strata, including Swedish-born strata with low income” as well as the particular high risk of loneliness among immigrants in nursing homes with lower income [20]. The study on Health, Aging and Retirement Transitions in Sweden collected data from 2015 to 2020 (March/April) of 1,071 adults born 1949 to 1955. Results showed that greater worry about financial consequences related to the COVID-19 pandemic was related to higher risk of loneliness [21].

Financial worry was only measured in 2020; not in previous waves. The Longitudinal Aging Study Amsterdam COVID-19 survey analyzed data of 1,089 Dutch adults aged 55 to 84 years in June and October 2020 [22]. The study created a COVID-19 exposure index which included two items on financial problems and job loss out of 35 items. Older adults in the top tercile of the COVID-19 exposure index reported higher risk of loneliness compared to the lowest tercile; the individual index items were not analyzed. A second Dutch study is based on responses of 1,679 Dutch adults age 65 and older using the Longitudinal Internet Studies for the Social Sciences (LISS) panel in the Netherlands in October/November 2019 and May 2020. Household income was a control measure and was not significantly associated with social or emotional loneliness in this sample [23].

The Hiroshima University’s Household Behavioral and Financial Survey in Japan surveyed 4,253 adults in February 2020 and February 2021 [24]. Higher household income was associated with lower risk of loneliness among adults age 65 and older in pre-pandemic 2020. Household assets were not associated with loneliness [24]. A year later, in 2021, the study finds a positive association of higher household income and higher risk of loneliness among the older adults; again no role of household assets.

Already prior to the Covid-19 pandemic, studies have identified a link between liquidity constraints and risk of loneliness in older age. A small number of studies documented that lower assets and lower income were associated with higher risk of loneliness among older adults [25–27], with the lowest income quartile or quintile of older adults more likely to report persistent risk of loneliness [28], while households with greater net wealth quintiles reported lower risk of loneliness [26, 29]. Linkages between older adults’ participation in the social life of their communities and loneliness have been established [for review, see 30, 31].

Taken together, international and U.S.-based data confirm a link between pandemic-related liquidity constraints and higher risk of loneliness. Financially vulnerable demographic groups, such as lower-income and lower-wealth groups, were also associated with higher risk of loneliness in most studies. The relative role of household income and access to savings has only been addressed in the Japanese study [24]. The limited understanding of the relative role of household income and savings is noteworthy because four in five retired older adults in the U.S. rely on their savings, in addition to Social Security retirement benefits [32].

We propose to examine the link between income, savings, and loneliness within the framework of the stress process theory [33]. This framework identifies liquidity constraints as a direct predictor of mental health outcomes, rather than one that is mediated by social support and psychological measures of personal control [34]. Based on the stress process framework, income as a measure of liquidity constraints has limitations because it does not reflect individuals’ ability to manage their money. As a complement measure to income, older adults’ ability to access savings to pay for a modest, unexpected expense of $400 from liquid savings has been used in other studies [35, 36]. An individuals’ emergency savings is a richer liquidity constraint measure because it captures vulnerability to income and expense shocks [35]. Our working hypothesis is that household savings are a stronger predictor of the risk of loneliness than household income for older adults because they reflect older households’ economic position more accurately.

Surveys conducted during the initial wave of the COVID-19 pandemic document that the majority of older adults took actions to avoid a COVID-19 infection in Spring 2020, even reporting to shelter in place prior to state orders [5, 37]. While the U.S. Centers for Disease Control and Prevention, health care providers, and aging institutions issued specific guidance for older adults that supported social distancing, staying at home, avoiding crowds, cruises, and non-essential air travel [38], little is known about the actual actions taken by older adults to respond to such a threat. For example, the National Health and Aging Trends Study asked older adults who reported experiencing financial difficulty due to the pandemic about the strategies they needed to manage financial difficulties. Results show that older adults who listed a higher number of strategies to manage financial difficulties also reported greater loneliness [13].

Similarly, only limited data are available about which interruptions of personal plans of older adults occurred in the initial wave in Spring 2020 [39]. An emphasis of research has been on describing the reduced socialization with friends and family as related to loneliness [4–6]. Little detailed information is available about other disruptions, such as reduced public transportation, closures of bars, restaurants, gyms, and travel restrictions in their association with loneliness. Based on the available research, our working hypothesis is that more actions taken and greater disruptions experienced by older adults are associated with a greater risk of loneliness. In addition, we expect responses to be relative to liquidity constraints. Following earlier studies, those with higher household incomes and wealth who experience more COVID-19-related actions and disruptions are expected to have a higher risk of loneliness [6, 19]. If government-imposed lockdown and stay-at-home orders restricted social contacts, the easing of the restrictions should have lowered levels of loneliness [40, 41]. However, the spring and early summer of 2020 remained a stressful time that may have limited gatherings despite the reopenings. First, the street demonstrations and protests in cities across the United States that initiated the “Black Lives Matter” movement deeply worried people about stepping back out into the public meeting spaces for weeks, dominated by news reports in the United States [42]. Older adults felt particularly restrained by these events because the fear of infection may have prevented them from voicing their concerns by participating in demonstrations [43].

Second, even as states reopened in May and June 2020, infections and deaths remained high, and those statistics were reported widely to the public. Milestones were emphasized, such as when the death toll reached 100,000 at the end of May 2020 [44]. These reports were highly distressing for all age groups, but possibly more for the population age 65 and older that suffered the highest death rates due to COVID-19 [45]. Third, during spring and early summer 2020, it became clear that COVID-19 was spreading globally, and the public discussion emphasized the fact that it may take years to develop effective vaccines. Taken together, we hypothesize that the growing realization about the long-term burden of COVID-19 might have eliminated the benefits of easing lockdown restrictions and resuming social relationships.

## 4. Methods

### 4.1 Data

We use data from three waves of the Data Foundation’s COVID Impact Survey, that targeted the adult population of the United States during the initial COVID-19 wave [46]. Data of a representative general population sample were collected by University of Chicago’s Nonpartisan and Objective Research Organization (NORC) using both area probability sampling through its AmeriSpeak Panel and address-based sampling methods. The survey was administered as a repeated cross-section of the population and was collected between April 20 to 26 (Wave 1), May 4 to 17 (Wave 2), and May 30 to June 8, 2020 (Wave 3). Institutional Review Board (IRB) approval was obtained by project director Jennifer Benz through NORC’s IRB at the University of Chicago (IRB00000967), IRB Protocol Number 20.04.10 with the exemption granted on 4/10/2020, exemption category 45 CFR 46.104(d)(2)(iii). Verbal informed consent was obtained prior to the commencement of the online and phone-based data collection, which was administered by NORC’s proprietary data collection platform.

We limited the sample to adults aged 65 and older, n = 7,149. This cut-off was chosen based on the available survey age categories, which used 10-year age ranges, and the common approach to use Medicare eligibility at age 65 as the lower threshold in aging studies [47]. The regression sample size was n = 5,664 (79.2%). Responses with missing values (n = 1,485) were dropped from the analysis, following common procedure [48], instead of using procedures such as full information maximum likelihood and multiple imputation, because more than half of missing values, 66 per cent, were from a single variable, the race variable. Race had the largest number of missing values for a total of 13.7 per cent (n = 978). Of those 978 cases, 967 cases were “removed for disclosure risk” (13.5%) by the data provider because it may be possible to identify respondents based on racial characteristics. As a result, the sample was biased toward White respondents. The second variable with a larger number of missing values was household income with 325 missing values (4.5%). All other measures had responses from at least 98.0 percent of respondents.

### 4.2 Sample description

This sample from Spring 2020 consisted of adults ages 65 and older, with the majority age 65 to 74 (70.3%), with slightly more female respondents compared to the population of older adults in general (52.3%; U.S. 2019: 55.4% [8]). The respondents with race-related data are mostly White (84.0%; U.S. 2019: 76% [8]), with Black respondents presenting the largest minority group (7.2%; U.S. 2019: 9% [8]), pointing to a slightly less racially diverse study sample than the population of older adults at large. The racial bias was due to the data provider’s practice to reduce disclosure risk, which compromised the race variables.

More than half of the sample had a college degree (52.0%; U.S. 2019: 33% [8]) and another third of this sample of older adults reports some college (32.0%), indicating a higher-educated sample than the general population age 65 and older. Survey respondents lived in households of 1 to 2 persons. The regions with the most respondents were South Atlantic (19.5%), East North Central (17.9%), and Mid-Atlantic (13.0%). About equal numbers of respondents were from each of the three April, May, and June 2020 survey waves (wave 1 = 33.1%, wave 2 = 38.7%, wave 3 = 29.1%). The majority of respondents were urban residents (78.6%), which reflects United States Census data that 80% of general population live in urban areas [49]. Very few respondents or their household members had COVID-19 in Spring 2020 (0.4%). The older adults in this sample reported on average 2.3 comorbidities, enrollment in an average of 2 health insurance plans, and had applied for or received an average of 1 to 2 forms of financial assistance.

Tables 1 and 2 contain descriptive statistics for this sample of older adults showing which groups experienced more loneliness. About 25.3% of respondents reported having felt lonely in the past 7 days (1–2 days: 17.2%; 3–4 days: 5.1%; 5–7 days: 3.0%). Lonely respondents in our sample of older adults differed from those who were not lonely in several important ways. Lonely respondents had significantly lower household incomes ($65,685 vs $70,402) and more often indicated difficulty handling a $400 unexpected financial emergency (31.3% vs 24.3%). They indicated much more often to put the $400 financial emergency on a credit card and pay it off over time (20.1% vs 14.0%) and being less able to pay it off in full at the next statement compared to respondents who did not indicate loneliness (52.8% vs 58.9%). Lonely respondents in our sample took a greater number of actions in response to COVID-19 (9.6 vs 8.7 actions) and had a greater number of personal plans changed or affected by COVID-19 restrictions (6.3 vs 5.5 plans).

**Table 1:** Descriptive statistics and means comparison tests for respondents who report loneliness and those who do not; focal measures.

Variables (Range)	Total	No loneliness	Loneliness	Means comparison
		Mean (SD), %	Mean (SD), %	p value
Loneliness (1–4)	1.36 (0.71)	1.00 (0.00)	2.43*** (0.69)	p<0.001
Liquidity constraints				
Annual household income (5,000–150,000)	$69,205 ($42,947)	$70,402 ($6,636)	$65,685*** ($1,112)	p<0.001
Lack emergency savings (0/1)	26.11%	24.34%	31.31%***	p<0.001
Put it on my credit card and pay it off in full at the next statement (0/1)	57.31%	58.90%	52.88%***	p<0.001
Use money currently in my checking or savings account or with cash (0/1)	55.06%	55.73%	53.09%	p = 0.082
Put it on my credit card and pay it off over time (0/1)	15.60%	14.07%	20.11%***	p<0.001
Use money from a bank loan or line of credit (0/1)	2.59%	2.36%	3.27%	p = 0.062
Borrow from a friend or family member (0/1)	2.45%	2.50%	2.29%	p = 0.654
Use a payday loan, deposit advance or overdraft (0/1)	0.68%	0.66%	0.76%	p = 0.683
Sell something (0/1)	1.95%	1.70%	2.71%*	p = 0.017
I would not be able to pay for it right now (0/1)	7.39%	7.07%	8.35%	p = 0.110
Actions taken (0–19)	8.99 (2.73)	8.78 (0.04)	9.62 (0.06)***	p<0.001
Plans changed (0–19)	5.72 (3.79)	5.51 (0.05)	6.33 (0.09)***	p<0.001
Reopened (0/1)	63.15%	63.63%	61.72%	p = 0.194
N (%)	5,664	4,227 (74.63%)	1,437 (25.37%)	

Note: * p<0.05, ** p<0.01, *** p<0.001

**Table 2:** Descriptive statistics and means comparison tests for respondents who report loneliness and those who do not; control measures.

Variables (Range)	Total	No loneliness	Loneliness	Means comparison
		Mean (SD), %	Mean (SD), %	p value
Health-related controls:				
Respondent had COVID-19 (0/1)	0.40%	0.40%	0.41%	p = 0.937
Household member had COVID-19 (0/1)	0.44%	0.44%	0.41%	p = 0.874
Number comorbidities (0–12)	2.39 (1.72)	2.27 (0.02)	2.74 (0.04)***	p<0.001
Financial controls:				
Number health insurance (0–7)	2.27 (0.96)	2.26 (0.01)	2.31 (0.02)*	p = 0.050
Number financial assistance (0–8)	1.44 (1.12)	1.40 (0.01)	1.55 (0.03)***	p<0.001
Socio-demographic controls:				
Age 75 plus (0/1)	29.64%	68.86%	74.73%***	p<0.001
Male (0/1)	47.63%	51.21%	37.09%***	p<0.001
Non-Hispanic White (0/1)	84.03%	83.18%	86.57%**	p = 0.002
Non-Hispanic Black (0/1)	7.22%	7.97%	5.01%***	p<0.001
Non-Hispanic Other (0/1)	4.29%	4.33%	4.18%	p = 0.803
Hispanic (0/1)	4.44%	4.52%	4.24%	p = 0.663
No HS diploma (0/1)	2.34%	2.20%	2.78%	p = 0.207
HS graduate or equivalent (0/1)	13.59%	13.84%	12.87%	p = 0.356
Some college (0/1)	32.02%	32.17%	31.59%	p = 0.683
BA or above (0/1)	52.03%	51.79%	52.75%	p = 0.528
Household size (1–6)	1.74 (0.01)	1.75 (0.01)	1.72 (0.02)	p = 0.156
Census region				
New England (0/1)	1.11%	1.18	0.90	p = 0.385
Mid-Atlantic (0/1)	13.04%	13.01	13.15	p = 0.891
EN Central (0/1)	17.90%	17.41	19.35	p = 0.098
WN Central (0/1)	9.78%	9.89	9.46	p = 0.639
S Atlantic (0/1)	19.52%	19.68	19.07	p = 0.611
ES Central (0/1)	5.06%	5.44	3.97%*	p = 0.027
WS Central (0/1)	9.51%	9.87	8.49%	p = 0.124
Mountain (0/1)	12.25%	12.37	11.90%	p = 0.636
Pacific (0/1)	11.79%	11.14	13.71%**	p = 0.009
Wave 1 4/20-26/2020 (0/1)	33.12%	33.07%	33.26%	p = 0.894
Wave 2 5/4-10/2020 (0/1)	37.72%	37.21%	39.25%	p = 0.169
Wave 3 6/1-8/2020 (0/1)	29.14%	29.71%	27.49%	p = 0.108
Rural residence (0/1)	5.19%	5.49%	4.31%	p = 0.083
Suburban residence (0/1)	16.20%	16.25%	16.08%	p = 0.874
Urban residence (0/1)	78.60%	78.26%	79.61%	p = 0.280
Survey language is English (0/1)	99.43%	99.43%	99.44%	p = 0.961
Survey mode is web (0/1)	86.51%	85.87%	88.37%*	p = 0.016
N (%)	5,664	4,227 (74.63%)	1,437 (25.37%)	

Note

* p<0.05

** p<0.01

*** p<0.001

Turning to control measures, lonely older adults in our sample reported a larger number of comorbidities (2.7 vs 2.2 comorbidities), health insurance or health coverage plans (2.3 vs 2.2 plans), and financial assistance applied for or received (1.5 vs 1.4 forms of assistance). Lonely respondents were more often over age 75 (74.7% vs 68.8%), female (62.9% vs 48.7%), and White rather than Black (5.0% vs 7.9%). The Pacific region had higher numbers of lonely older adults (13.7% vs 11.1%), and lonely older adults more often chose to take the survey by web rather than phone (88.3% vs 85.8%).

### 4.3 Measures

#### 4.3.1 Dependent variable—Loneliness

Loneliness was measured with a single question, “In the past 7 days, how often have you felt lonely?”, an approach which has been used in other loneliness research [28]. Response options were taken from the Center for Epidemiologic Studies Depression Scale [50] and included four categories: not at all or less than 1 day (coded as 1); 1–2 days (coded as 2); 3–4 (coded as 3) days; or 5–7 days (coded as 4). We used this original coding in our data analysis. For robustness checks, we also used a binary measure with “not at all or less than 1 day” coded as 0 (otherwise as 1); and an upper-level coding with “not at all or less than 1 day” (coded as 1); 1–2 days (coded as 2); 3–4 days (coded as 4); or 5–7 days (coded as 7).

#### 4.3.2 Focal predictor variables—Liquidity constraints

The focal predictor variables were two measures of liquidity constraints: annual household income and lack of emergency savings. The annual household income measure (under $10,000 = 1; $10,000 to under $20,000 = 2; $20,000 to under $30,000 = 3; $30,000 to under $40,000 = 4; $40,000 to under $50,000 = 5; $50,000 to under $75,000 = 6; $75,000 to under $100,000 = 7; $100,000 to under $150,000 = 8; $150,000 and higher = 9) was coded using the scale midpoints and, for the top category, the lower bound. Categorical, lower and upper bound coding was used for robustness tests.

We measured lack of emergency savings with a widely used question [51], inquiring, “Suppose you have an unexpected expense that costs $400. Based on your current financial situation, how would you pay for this expense? If you would use more than one method to cover this expense, please select all that apply.” The eight response options included “Use money currently in my checking or savings account or with cash” (= 1); “Put it on my credit card and pay it off over time” (= 2); “Put it on my credit card and pay it off in full at the next statement” (= 3); “Use money from a bank loan or line of credit” (= 4); “Borrow from a friend or family member” (= 5); “Use a payday loan, deposit advance or overdraft” (= 6); “Sell something” (= 7); “I would not be able to pay for it right now” (= 8). Response options included yes (coded as 1), no (coded as 0), don’t know (coded as 0); refusals and skipped responses are coded as missing. The top-three selected items of the list were: “Use money currently in my checking or savings account or with cash” (57.6%); “Put it on my credit card and pay it off in full at the next statement” (55.6%); and “Put it on my credit card and pay it off over time” (15.2%).

Following the coding suggested by the Federal Reserve [52], we created a binary measure for which items 2, 4, 5, 6, 7, 8 are coded as lacking emergency savings (= 1), and items 1 and 3 are coded as having emergency savings (= 0).

#### 4.3.3 Focal predictor variables—Actions taken and personal plans changed in response to COVID-19

We tested the relationship between liquidity constraints and loneliness by examining actions taken and personal plans changed as a result of the initial COVID-19 wave. Actions taken in response to COVID-19 were recorded with the question, “Which of the following measures, if any, are you taking in response to the coronavirus?” Response options included yes (coded as 1), no (coded as 0), don’t know (coded as 0) to 19 items, see S2 Table (available in S1 File). The top-three selected items were “Washed or sanitized hands” (96.9%), “Kept six feet distance from those outside my household” (94.5%), and “Worn a face mask” (89.9%). Refusals and skipped responses were coded as missing.

The second measure, personal plans changed as a result of COVID-19, was inquired with the question, “In the past 7 days, have your personal plans been changed or affected by the following types of restrictions, or not?” Response options included yes (coded as 1), no (coded as 0), not sure (coded as 0) to again 19 items, see S3 Table (available in S1 File). The top-three selected items were “Domestic travel restrictions or bans” (63.9%), “International travel restrictions or bans” (35.3%), “Ban on gatherings of 250 people or more” (30.5%).

For Research Questions 4, as a binary measure of reopening after lockdown, state public health department orders were reviewed to create a binary measure of whether a state had eased lockdown restrictions by the survey date of any of the three weeks in which the COVID Impact Survey was collected, which was coded as 1. States with lockdown restrictions still in effect as of the survey dates were coded as 0. If the state eased restrictions during the week of the survey, the state was coded as reopened. The date that retail stores were allowed to open is used to indicate that the lockdown is eased, see S4 Table (available in S1 File) for an overview of the states. Missouri and Texas opened during the first wave, and thus were coded as reopened for entire study period. All states except for California, Minnesota, and New York opened before or during the second wave, coded as reopened for waves 2 and 3. Minnesota and New York reopened before wave 3, coded as reopened for wave 3.

#### 4.3.4 Control variables

Health-related control variables included binary measures of the respondent diagnosed with COVID-19 (yes = 1, no = 0), a household member diagnosed with COVID-19 (yes = 1, no = 0), and a family member or close friend died from COVID-19 (yes = 1, no = 0). We controlled for 13 common comorbidities with a summed measure based on the question, “Has a doctor or other health care provider ever told you you have any of the following?” with the top three items being “High blood pressure or hypertension” (55.1%); “Allergies” (42.9%); “Overweight or obesity” (33.5%).

Financial controls included eight health insurance coverage options with a summed measure based on the question, “Are you currently covered by any of the following types of health insurance or health coverage plans?” with the top three items being Medicare (92.2%); Other health insurance or health coverage plan (42.0%); Insurance purchased directly from an insurance company by you or another family member (33.8%). We also controlled for 12 financial assistance options with a summed measure based on the question, “In the past 7 days, have you either received, applied for, or tried to apply for any of the following forms of income or assistance, or not?” with the top three items being Social Security (69.3%); any kind of government health insurance or health coverage plan including Medicaid, Medical Assistance or Medicare (44.2%); other aid from the government (10.6%).

Socio-demographic control variables included gender (male = 1, female = 0), age (65–74 = 0, 75 and older = 1), race (non-Hispanic White = 1 (omitted), non-Hispanic Black = 2, non-Hispanic other = 3, Hispanic = 4), educational attainment (no high school diploma = 1 (omitted), high school gradute or equivalent = 2, some college = 3, BA or above = 4), household size (cont.), census region (New England (omitted), Mid-Atlantic, East North Central, West North Central, South Atlantic, East South Central, West South Central, Mountain, Pacific), survey wave (April 20–26 = 1 (omitted), May 4–10 = 2, June 1–8 = 3), population density (rural = 1 (omitted), suburban = 2, urban = 3), survey language (English = 1; Spanish = 0); and survey mode (web interview = 1, phone interview = 0).

### 4.4 Empirical strategy

We assessed Research Question 1—do household income and emergency savings predict loneliness among older adults during the initial COVID-19 wave—by using OLS regression to regress loneliness on annual household income and emergency savings, controlling for the health-related, financial, and socio-demographic characteristics.

For Research Question 2, the measure “lack of emergency savings” was replaced with the eight individual items of the scale and the OLS regression analysis was repeated. For Research Question 3, two variables were added to the equation, the number of personal actions taken to avoid a COVID-19 infection and the number of personal plans changed due to COVID-19. We used a two-step analysis approach, with the base specification including the two COVID-19 actions taken and plans changed variables and the interaction specification including the interaction terms of the two COVID-19 actions taken and plans changed measures and the financial liquidity measures. For Research Question 4, we examined whether the focal results had changed if respondents lived in states that reopened during the study period. We examined this question by adding the reopening variable and with two subsample regressions for respondents who lived in states that reopened during the study period (n = 3,577) and those who lived in states that did not reopen during the study period (n = 2,087).

## 5. Results

### 5.1 Research Question One: Role of income and emergency savings for loneliness

Research Question 1 assesses the relationship between liquidity constraints and loneliness during the initial wave of COVID-19. Table 3, Column 1 contains our main results; full results are in S5 Table (available in S1 File). We regress loneliness on household income and lack of emergency savings, controlling for health-related, financial, and socio-demographic measures. The OLS regression results indicate a strong association of lack of emergency savings with loneliness at p<0.001. The data do not support an association between household income and loneliness in this sample of older adults during the initial COVID-19 wave (p = 0.114). Categorical coding of the income variable and continuous coding at the lower and upper bounds of the income category brackets does not change this result, see S6 Table (available in S1 File).

**Table 3:** Coefficients of OLS regression of loneliness on liquidity constraints, actions taken and plans changed, and interaction terms.

Variables	(1) Loneliness	(2) Loneliness	(3) Loneliness	(4) Loneliness
	Coef. (S.E.)	Coef. (S.E.)	Coef. (S.E.)	Coef. (S.E.)
Annual household income	-0.004 (0.002) p = 0.114	-0.003 (0.002) p = 0.186	-0.007*** (0.002), p = 0.008	-0.005 (0.008) p = 0.528
Lack emergency savings	0.130*** (0.023) p<0.001	--	0.120*** (0.023) p<0.001	0.152* (0.077) p = 0.050
Individual items:				
Put it on my credit card and pay it off in full at the next statement		-0.053* (0.023) p = 0.024		
Use money currently in my checking or savings account or with cash		-0.008 (0.021) p = 0.674		
Put it on my credit card and pay it off over time		0.124*** (0.029) p<0.001		
Use money from a bank loan or line of credit		0.038 (0.061) p = 0.355		
Borrow from a friend or family member		-0.060 (0.065) p = 0.355		
Use a payday loan, deposit advance or overdraft		0.078 (0.117) p = 0.502		
Sell something		0.160* (0.072) p = 0.027		
I would not be able to pay for it right now		0.081 (0.045) p = 0.073		
Actions taken			0.020*** (0.003) p<0.001	0.021* (0.008) p = 0.010
Plans changed			0.011*** (0.002) p<0.001	0.013** (0.005) p = 0.023
Interaction terms:				
Actions taken * Income				0.000 (0.0009) p = 0.994
Plans changed * Income				-0.0003 (0.0006) p = 0.627
Actions taken * Lack emergency savings				-0.003 (0.008) p = 0.693
Plans changed * Lack emergency savings				-0.0002 (0.006) p = 0.974
Control measures:				
Health-related measures	Yes	Yes	Yes	Yes
Financial, socio-demographic measures	Yes	Yes	Yes	Yes
F test (df)	9.50*** (30)	8.18*** (37)	11.15*** (32)	9.92*** (36)
Adj. R-squared	0.048	0.044	0.059	0.053
Observations	5,664	5,664	5,664	5,664

Note

* p<0.05

** p<0.01

*** p<0.001

Among control measures, a higher number of health comorbidities, higher access to financial assistance, and urban residence are associated with higher risk of loneliness. Respondents in the older 75 plus age group, male, non-Hispanic Black (compared to non-Hispanic White respondents), respondents and those with a high school education only had a lower risk of loneliness. These associations remain consistent across the specifications shown in Table 4.

### 5.2 Research Question Two: Types of liquidity constraints related to loneliness

Research Question 2, which examines the relative role of the eight emergency savings items, is addressed in Table 3, Column 2. A higher level of loneliness is strongly associated with respondents covering a $400 financial emergency by putting the expense on a credit card and paying it off over time, at p<0.001. In addition, loneliness is higher if respondents indicated the need to sell something to cover a $400 financial emergency, at p = 0.027; this response is only selected by 1.95 per cent. In contrast, if respondents are able to pay off the $400 in full at the next statement, the association with loneliness is negative, at p = 0.024.

### 5.3 Research Question Three: Role of actions taken in response to COVID-19 and personal plans changed

Table 3, Columns 3 and 4 show the results for Research Question 3, which addresses the role of personal actions taken and personal plans changed due to COVID-19. We examine this research question by interacting the liquidity measures (income and lack of emergency savings) with the two COVID-19 actions taken and plans changed variables, controlling for the health-related, financial and socio-demographic characteristics. The main effects model in Column 3 indicates a significant positive association of actions taken and personal plans changed with loneliness, both at p<0.001. The two liquidity measures, income and emergency savings are also associated with loneliness in this specification, in the expected direction.

In Column 4 of Table 3, we add four interaction terms to the specification: the income and savings measures each interacted with actions taken and personal plans changed, respectively. The interaction terms are not significant at p<0.05. This finding indicates that income level and access to emergency savings do not influence the relationship between actions taken and personal plans changed as a result of the initial COVID-19 wave. This is a key finding and supports the notion that in the early months of the COVID-19 pandemic, loneliness associated with the actions taken to avoid COVID-19 and personal plans changed was experienced across all socio-economic groups of older adults in our sample in similar ways, regardless of income levels and wealth. We checked the robustness of our empirical approach by testing a binary measure of loneliness and an upper-level coding, see S7 Table (available in S1 File). These results are consistent with the focal results in direction and magnitude.

To provide a more detailed understanding of these findings, we conducted subsample analysis for particularly financially vulnerable groups of older adults in our sample: women (n = 2,966; 52.4% of the sample), younger age group of 65–74 years of age (n = 3,985, 70.4% of the sample), and minority (non-white) older adults (n = 904; 16.0% of the sample). These respondent characteristics emerged as significant predictors of loneliness in the main specification. The subsample regressions, shown in S8 Table (available in S1 File), align with the full-sample results. We do not find significant interaction effects of actions taken and plans changed with the two liquidity measures in the three subsample regressions.

### 5.4 Research Question Four: Role of easing lockdown restrictions

Table 4 contains results for Research Question 4, examining the role of states easing economic and social restrictions. We do not find an association between reopened status and household income, emergency savings, or loneliness, respectively. In subsample regressions, see Table 5, results show that in states that reopened quickly, lack of emergency savings rather than income is associated with loneliness, while in states that had not reopened by June 8, 2020, lower household income is more strongly associated with loneliness than lacking emergency savings.

**Table 4:** Coefficients of regression of loneliness and liquidity constraints on reopened status in a state.

Variables	(1) Annual household income	(2) Lack emergency savings	(3) Loneliness
	OLS Coef. (S.E.)	Logistic Coef. (S.E.)	OLS Coef. (S.E.)
Reopened	-0.160 (0.186) p = 0.391	0.216 (0.130) p = 0.098	0.002 (0.036) p = 0.950
Annual household income	--	--	-0.007** (0.002) p = 0.008
Lack emergency savings	--	--	0.120*** (0.023) p<0.001
Actions taken	0.107 (0.019) p<0.001	0.004 (0.013) p = 0.714	0.020*** (0.003) p<0.001
Plans changed	0.070 (0.013) p<0.001	0.013 (0.009) p = 0.140	0.011*** (0.002) p<0.001
Survey wave 2, 5/4-10/2020	0.124 (0.190) p = 0.512	-0.214 (0.132) p = 0.105	0.011 (0.036) p = 0.752
Survey wave 3, 6/1-8, 2020	-0.021 (0.195) p = 0.912	-0.105 (0.134) p = 0.435	-0.006 (0.037) p = 0.867
Control measures:			
Health-related measures	YES	YES	YES
Financial, socio-demographic measures	YES	YES	YES
F test/Chi2 (df)	78.63*** (31)	Chi2 = 924*** (31)	10.81*** (33)
Adj./Pseudo R-squared	0.298	0.142	0.054
Observations	5,664	5,664	5,664

Note

* p<0.05

** p<0.01

*** p<0.001

**Table 5:** Coefficients of OLS regression of loneliness on liquidity constraints, actions taken and plans changed, and interaction terms: Subsamples with and without reopening.

Variables	(1) Loneliness (Subsample: Reopened during study period)^a^	(2) Loneliness (Subsample: Reopened during study period)^a^	(3) Loneliness (Subsample: Not reopened during study period)^a^	(4) Loneliness (Subsample: Not reopened during study period)^a^
		Coef. (S.E.)		Coef. (S.E.)
Annual household income	-0.002 (0.003) p = 0.466	-0.003 (0.010) p = 0.725	-0.014** (0.004) p = 0.001	-0.008 (0.013) p = 0.522
Lack emergency savings	0.145*** (0.029) p<0.001	0.120 (0.095) p = 0.210	0.072 (0.039) p = 0.069	0.192 (0.134) p = 0.152
Actions taken	0.019*** (0.004) p<0.001	0.014 (0.010) p = 0.159	0.022*** (0.006) p<0.001	0.032* (0.013) p = 0.018
Plans changed	0.013*** (0.003) p<0.001	0.018* (0.005) p = 0.016	0.008* (0.004) p = 0.044	0.005 (0.010) p = 0.614
Interaction terms:				
Actions taken * Income	--	0.0052 (0.001) p = 0.645	--	-0.0007 (0.001) p = 0.609
Plans changed * Income	--	-0.0006 (0.0008) p = 0.450	--	-0.0001 (0.001) p = 0.853
Actions taken * Lack emergency savings	--	0.004 (0.010) p = 0.660	--	-0.018 (0.014) p = 0.210
Plans changed * Lack emergency savings	--	-0.003 (0.007) p = 0.676	--	0.007 (0.010) p = 0.440
Control measures:				
Health-related measures	Yes	Yes	Yes	Yes
Financial, socio-demographic measures	Yes	Yes	Yes	Yes
F test (df)	8.05*** (32)	7.17*** (36)	3.71*** (36)	3.71*** (36)
Adj. R-squared	0.059	0.058	0.044	0.044
Observations	3,577	3,577	2,087	2,087

Note

* p<0.05

** p<0.01

*** p<0.001

^a^Reopened variable: Missouri and Texas opened during first wave, set as reopened for entire study period; All states except for California, Minnesota, & New York opened before or during the second wave, set as reopened for waves 2 and 3; Minnesota and New York reopened before wave 3, set as reopened for wave 3

## 6. Limitations

Several study limitations should be acknowledged. First, the study examines only the initial COVID-19 wave using data from April, May and June 2020. The findings cannot be generalized to the later waves of the COVID-19 pandemic. In addition, the three waves of survey data used in the current study are cross-sectional and do not allow for longitudinal data analysis. We controlled for survey wave to at least account for potential time-related response differences.

Second, we use a single-item, categorical measure of loneliness due to data collection constraints, rather than a scale composed of several items. Further, our loneliness variable is not an evidence-based measure. This is a common approach; five of the 14 studies in our literature review used a single item survey question [6, 13, 17, 20, 24]. Five studies used selected items from the UCLA Loneliness Scale [53–55], most often the three-item version [12, 14, 19, 21, 24]. Two studies used the three-item scale from Hughes et al. [15, 16, 56] and three studies used other scales. It has been noted that findings based on single-item measures tend to replicate findings based on multi-item measures [57, 58]. This general statement is not true for Khan and Kadoya [24] in our literature review, which provides results for both the UCLA and a single-item loneliness scale. In this study the single item direct measure tends to be less strongly associated with the predictors, indicating more conservative measurement. The HRS, for example, also uses a single-item loneliness question in its core questionnaire (Question D114) [59].

Third, the sample is biased toward higher educated, White respondents. A 19 percentage point larger portion of respondents had a college degree compared to the general population of adults age 65 and older (this study: 52.0%; U.S. 2019: 33% [8]). This is likely due to the online-based data collection and a lack of funding to oversample older adults with lower educational attainment. Race has the largest number of missing values in this data set, a total of 13.7 per cent. Almost all missing values, 13.5%, were “removed for disclosure risk” by the data provider, resulting in a 8 percentage-point bias toward White respondents (this study: 84.0%; U.S. 2019: 76% [8]) and a smaller sample of Black respondents (this study: 7.2%; U.S. 2019: 9% [8]). This sample bias should be kept in mind for the interpretation of the results. We cannot make conclusions about the prevalence of liquidity constraints, actions taken and plans changed for the general population of older adults in the U.S. thus limiting the generalizability of the findings.

Fourth, data are self-reported and their validity cannot be verified, as is typically the case in survey research. Relatedly, sample selection bias is a concern in survey research. The approach taken in this data collection effort that combines area probability sampling and address-based sampling methods is an advanced, proven approach for limiting selection bias [60].

## 7. Discussion

This study adds new knowledge about the role of liquidity constraints for loneliness among older adults during the initial COVID-19 wave [15]. First, we find for our sample of older adults that lack of emergency savings—but not household income [6]—was associated with higher levels of loneliness from April to June 2020. Household income did not protect the older adults in our sample against loneliness during the early months of the COVID-19 pandemic, as has been described in pre-COVID literature [25]. This finding extends insights from pre-COVID-19 studies that for older adults, wealth is a stronger correlate of health outcomes than income [61, 62] and documents that the ability to rely on emergency savings was beneficial during the initial COVID-19 wave [15]. Future research in loneliness should explore further the approaches of older adults for meeting financial emergencies. Fixed incomes coupled with a greater vulnerability to having plans changed and needing to take action might place a greater emphasis on the relationship of limited liquid assets and the risk of loneliness.

Our second key finding is the role of having to rely on credit card debt for older adults’ risk of loneliness. We find that covering a $400 financial emergency by putting it on a credit card and paying it off over time was associated with higher risk of loneliness while those who were able to pay off the $400 in full at the next credit card statement showed lower levels of loneliness. Already prior to the COVID-19 pandemic, credit card debt has been shown to be particularly strongly associated with financial stress among older adults [63], and financial stress with higher levels of loneliness among older adults [64]. The current study documents the focal role of having to rely on credit card debt in financial emergencies for the understanding of loneliness in older age during the COVID-19 pandemic and points to the need to account for debt measures in the assessment of loneliness in older age, a perspective that has received limited attention.

The third study finding provides new insights on the role of actions taken by older adults to avoid a COVID-19 infection and the role of personal plans changed due to COVID-19. A key finding of the study is that the association of these COVID-19 factors with loneliness holds across economic groups in our sample. Whether or not older adults in the sample had emergency savings did not influence the strong positive relationship of loneliness with actions taken by these older adults and personal plans changed in the lives of these older adults. The same finding holds for different levels of household income. This study documents that the COVID-19 pandemic uniquely reached across economic strata of older adults in our sample to expose this population group to loneliness.

The fourth focus of this study was on the role of easing lockdown restrictions on the associations of loneliness with liquidity constraints, personal actions taken, and plans changed, a perspective that has not been provided in earlier research [19, 20]. Despite the impactful role of lockdowns on individuals and families, our data showed that the strong association of loneliness with liquidity constraints and COVID-19 measures did not ease for respondents in our sample after lockdowns were lifted. This finding points to longer-lasting implications of loneliness experienced due to liquidity constraints.

In conclusion, the current study provides two new aspects for the understanding of predictors of loneliness in older age. First, findings point to the role of limited savings, especially the intention to use revolving credit in case of unexpected expenses, rather than income as associated with loneliness. This finding aligns with research about the association of credit card debt with financial worry [63, 65]. It contributes to the understanding of predictors of loneliness by pointing to the role of assets rather than income for the experience of loneliness in older age [28]. Second, findings indicate that during the public health emergency that characterized the initial wave of COVID-19, the penetration of loneliness occurred across economic groups of older adults. Actions taken and plans changed by older adults were related to loneliness–regardless of the older adults’ economic situation. It is expected that this finding is unique to the initial months of the COVID-19 pandemic.

## Supporting information

S1 File (DOCX)
