# Supplementary material for: The association of financial resources and loneliness among older adults during a state of emergency
Source: PLoS One. 2025 Jan 9;20(1):e0314042. doi: 10.1371/journal.pone.0314042 (PMC11717192; doi:10.1371/journal.pone.0314042)
Supplement: S1 File — (DOCX) [file pone.0314042.s001.docx]

**The association of financial resources and loneliness**

**during a state of emergency**

## Supporting Information

S1 Table. Scoping review of the literature

| **Authors** | **Sample** | **Results** |
| --- | --- | --- |
| **Studies using data from the United States** | | |
| 1-O’Shea et al. [1]  “Loneliness Among US Adults Aged ≥55 Early in the COVID-19 Pandemic: Findings From the COVID-19 Coping Study” | Location: US  Name: COVID-19 Coping Study  Sample size, age: 6,938 aged ≥55  Data collected: April 2 through May 31, 2020  Loneliness: 3-item UCLA Loneliness Scale [2] | Individuals whose employment was adversely impacted during the pandemic had higher risk of loneliness than those whose employment was unaffected. Retired individuals had lower prevalence of loneliness than those who were employed pre-pandemic. |
| 2-Samuel et al. [3]  “The effect of COVID-19 pandemic-related financial challenges on mental health and well-being among US older adults” | Location: US  Name: National Health and Aging Trends Study  Sample size, age: 3,257 aged ≥65  Data collected: Interview 2019, supplemental survey June-October 2020.  Loneliness: Single-item frequency of loneliness during COVID-19 (rarely or never, some days, most days, every day) | Income decline and financial difficulty during the COVID-19 pandemic associated with higher risk of loneliness. Higher number of strategies needed to manage financial difficulties was associated with loneliness. |
| 3-Choi et al. [4]  “COVID-19 Social Distancing Measures and Loneliness Among Older Adults” | Location: US  Name: Understanding America Study COVID-19 Survey  Sample size, age: 3,253 aged ≥50  Data collected: April 29 to May 26, 2020  Loneliness: Single-item frequency of loneliness in past 7 days (not at all or less than 1 day, 1-2 days, 3-4 days, 5-7 days) | Social distancing measures were associated with higher risk of loneliness. Limiting close contact with people living together has higher risk of loneliness for males, non-Hispanic Whites, higher education and higher income individuals. |
| 4-Sams et al. [5]  “Understanding Psychological Distress and Protective Factors Amongst Older Adults During the COVID-19 Pandemic” | Location: US  Name: Survey given on crowdsourcing platforms Prolific and MTurk  Sample size, age: 501 60+ years old  Data collected: June 16-25, 2020  Loneliness: 3-item UCLA Loneliness Scale [2] | Individuals experiencing financial instability and lower socio-economic status had higher risk of loneliness. |
| 5-Polenick et al. [6] “Loneliness During the COVID-19 Pandemic Among Older Adults With Chronic Conditions” | Location: Michigan and 33 other US states  Name: Anonymous online survey  Sample size, age: 701 aged ≥50 with chronic conditions  Data collected: May 14 - July 9, 2020  Loneliness: Adapted 3-item loneliness scale of Hughes et al. [7] | Older adults with greater financial strain, higher anxiety, and more worry about COVID-19 had higher risk of loneliness. |
| **Studies using data from Canada, Europe, and Japan** | | |
| 6-Bierman et al. [8]  “Mattering and Self-Esteem as Bulwarks Against the Consequences of Financial Strain for Loneliness in Later Life: Differentiating Between- and Within-Person Processes” | Location: Canada  Name: Caregiving, Aging, and Financial  Experiences (CAFE) study  Sample size, age:  n=4010 pre survey, n=2,420 post survey, aged 65-85  Data collected: fall 2021 & fall 2022  Loneliness: 3-item loneliness scale of Hughes et al. [7] | Financial strain was associated with higher risk loneliness for older adults. |
| 7-Lee [9]  “Subjective Well-being and Mental Health During the Pandemic Outbreak: Exploring the Role of Institutional Trust” | Location: 27 European countries  Name: Eurofound COVID-19 cross-sectional survey  Sample size, age: 31,757 individuals aged 50+  Data collected: April 2020  Loneliness: Single-item frequency of loneliness in past 2 weeks (all of the time to at no time); part of a 3-item psychological distress scale | Worsening finances, difficulty paying for basic needs, and perceived employment insecurity were associated with higher risk of psychological distress. |
| 8-Mansfield et al. [10]  “Examining the Interrelationships between Social Isolation and Loneliness and Their Correlates among Older British Adults before and during the COVID-19 Lockdown: Evidence from Four British Longitudinal Studies” | Location: U.K.  Name: Four longitudinal studies: 1946 NSHD, 1958 NCDS, 1970 BCS, & ELSA.  Sample size, age: 12,129 ages 50+  Data collected: 2 waves prior to pandemic, one wave in early months of COVID-19 pandemic  Loneliness: Different scales in pre-pandemic waves were coded into a lonely/not lonely binary; all four studies used 3-item UCLA Loneliness Scale [2] in the pandemic waves | Individuals in economically precarious situations had higher risk of loneliness and social isolation both before and during the COVID-19 pandemic. Loneliness predictors remained consistent throughout the study time period. Women, unemployed individuals, non-homeowners, individuals with chronic illness, and individuals experiencing financial stress had higher risk of loneliness. |
| 9-Zaninotto et al. [11] “Immediate and Longer-Term Changes in the Mental Health and Well-being of Older Adults in England during the COVID-19 Pandemic” | Location: England  Name: English Longitudinal Study of Ageing  Sample size, age: 5,146 aged 52+  Data collected: pre-pandemic in 2018 & 2019, during pandemic in June/July 2020 and November/December 2020  Loneliness: 3-item UCLA Loneliness Scale [2] and single-item frequency of loneliness | Increase in loneliness was smaller for individuals in the lowest wealth group compared to the highest; however individuals in lowest wealth group had higher levels of loneliness before and during the COVID-19 pandemic. |
| 10-Gustafsson et al. [12]  “Intersectional inequalities in loneliness among older adults before and during the early phase of the COVID-19 pandemic: A total population survey in the Swedish eldercare setting” | Location: Sweden national  Name: Elderly Care Survey  Sample size, age: 205,529 home care and nursing home residents >65 years of age  Data collected: Two waves collected in 2019 and 2020  Loneliness: Single item, “Does it happen that you are troubled by loneliness?” (yes, no) | The highest risk of loneliness included low-income individuals, women, oldest adults, and immigrants. Older adults in nursing homes had highest risk for loneliness following the onset of the COVID-19 pandemic. |
| 11-van Tilburg et al. [13]  “Loneliness and Mental Health During the COVID-19 Pandemic: A Study Among Dutch Older Adults” | Location: The Netherlands  Name: Longitudinal Internet Studies for Social Sciences (LISS)  Sample size, age: 1,679 Dutch community-dwelling participants aged 65–102 years  Data collected: October/November 2019 and May 2020  Loneliness: de Jong Gierveld  short scales for emotional and social loneliness [14] | Higher risk of loneliness for older adults through the COVID-19 pandemic. Personal losses and worries about the COVID-19 pandemic associated with higher risk in emotional loneliness. Income is control measure, insignificant throughout. |
| 12-Kivi et al. [15]  “Up and About: Older Adults' Well-being during the COVID-19 Pandemic in a Swedish Longitudinal Study” | Location: Sweden  Name: HEARTS Study  Sample size, age: 1,071 older adults born 1949–1955 (age 60–66 at baseline)  Data collected: 2015 to 2020 (March 26 to April 2, 2020)  Loneliness: Items 2, 5, 11, and 14 of UCLA Loneliness Scale [16] | Greater worry about finances during the COVID-19 pandemic was associated to higher risk of loneliness. |
| 13-Hoogendijk et al. [17]  “Longitudinal Aging Study Amsterdam COVID-19 exposure index: a cross-sectional analysis of the impact of the pandemic on daily functioning of older adults” | Location: The Netherlands  Name: Longitudinal Aging Study Amsterdam (LASA) COVID-19  Sample size, age: 1,089 adults aged 55-84  Data collected: 9 June 2020-8 October 2020  Loneliness: De Jong Gierveld Loneliness scale [18] | Study uses a COVID-19 exposure index, a 35-item index, including economic measures. Scores in the highest tertile of exposure index had higher risk of loneliness. |
| 14-Khan and Kadoya [19]  “Loneliness during the covid-19 pandemic: A comparison between older and younger people” | Location: Japan  Name: Japan Household Behavioral and Financial Survey  Sample size, age: 4,253 individuals 21-86 (mean 50.3) years of age  Data collected: first wave online survey 20 to 25 February 2020, second wave of survey  19 to 26 February 2021  Loneliness: 3-item UCLA Loneliness Scale [2] and single-item frequency of loneliness | Household income and financial satisfaction were associated with higher risk of loneliness for older adults. |

References for the S1 Table:

1. O’Shea BQ, Finlay JM, Kler J, Joseph CA, Kobayashi LC (2021) Loneliness Among US Adults Aged ≥55 Early in the COVID-19 Pandemic: Findings From the COVID-19 Coping Study. Public Health Reports 136: 754-764.

2. Russell DW (1996) UCLA Loneliness Scale (version 3): Reliability, validity, and factor structure. Journal of Personality Assessment 66: 20-40.

3. Samuel LJ, Dwivedi P, Hladek M, Cudjoe TKM, Drazich BF, et al. (2022) The effect of COVID-19 pandemic-related financial challenges on mental health and well-being among US older adults. Journal of the American Geriatrics Society 70: 1629-1641.

4. Choi EY, Farina MP, Wu Q, Ailshire J (2022) COVID-19 Social Distancing Measures and Loneliness Among Older Adults. The Journals of Gerontology: Series B 77: e167–e178.

5. Sams N, Fisher DM, Mata-Greve F, Johnson M, Pullman MD, et al. (2021) Understanding Psychological Distress and Protective Factors Amongst Older Adults During the COVID-19 Pandemic. American Journal of Geriatric Psychiatry 29: 881-894.

6. Polenick CA, Perbix EA, Salwi SM, Maust DT, Birditt KS, et al. (2021) Loneliness During the COVID-19 Pandemic Among Older Adults With Chronic Conditions. Journal of Applied Gerontology 40: 804-813.

7. Hughes ME, Waite LJ, Hawkley LC, Cacioppo JT (2004) A short scale for measuring loneliness in large surveys: Results from two population-based studies. Research on Aging 26: 655-672.

8. Bierman A, Upenieks L, Lee Y, Mehrabi F (2024) Mattering and Self-Esteem as Bulwarks Against the Consequences of Financial Strain for Loneliness in Later Life: Differentiating Between- and Within-Person Processes. Research on Aging 46: 241-257.

9. Lee S (2022) Subjective Well-being and Mental Health During the Pandemic Outbreak: Exploring the Role of Institutional Trust. Research on Aging 44: 10-21.

10. Mansfield R, Gessa GD, Patel K, McElroy E, Wels J, et al. (2024) Examining the Interrelationships Between Social Isolation and Loneliness and Their Correlates Among Older British Adults Before and During the COVID-19 Lockdown: Evidence From Four British Longitudinal Studies. Innovation in Aging 8: 1-13.

11. Zaninotto P, Iob E, Demakakos P, Steptoe A (2022) Immediate and Longer-Term Changes in the Mental Health and Well-being of OlderAdults in England During the COVID-19 Pandemic. JAMA Psychiatry 79: 151-159.

12. Gustafsson PE, Fonseca-Rodríguez O, Nilsson I, Sebastian MS (2022) Intersectional inequalities in loneliness among older adults before and during the early phase of the COVID-19 pandemic: A total population survey in the Swedish eldercare setting. Social Science and Medicine 314: 115447.

13. van Tilburg TG, Steinmetz S, Stolte E, van der Roest H, de Vries DH (2021) Loneliness and Mental Health During the COVID-19 Pandemic: A Study Among Dutch Older Adults. The Journals of Gerontology: Series B 76: e249–e255.

14. De Jong Gierveld J, Van Tilburg T (2010) The de Jong Gierveld short scales for emotional and social loneliness: Tested on data from 7 countries in the UN generations and gender surveys. European Journal of Ageing 7: 121-130.

15. Kivi M, Hansson I, Bjälkebring P (2021) Up and About: Older Adults’ Well-being During the COVID-19 Pandemic in a Swedish Longitudinal Study. Journals of Gerontology: Psychological Sciences 76: e4-e9.

16. Russell D, Peplau LA, Cutrona CE (1980) The revised UCLA Loneliness Scale: Concurrent and discriminant validity evidence. Journal of Personality and Social Psychology 39: 472-480.

17. Hoogendijk EO, Schuster NA, Tilburg TGv, Schaap LA, Suanet B, et al. (2022) Longitudinal Aging Study Amsterdam COVID-19 exposure index: a cross-sectional analysis of the impact of the pandemic on daily functioning of older adults. BMJ Open 12: e061745.

18. Van Tilburg TG, De Jong Gierveld J (1999) Cesuurbepaling van de eenzaamheidsschaal [Reference standards for the loneliness scale]. Tijdschrift voor Gerontologie en Geriatrie 30: 158-163.

19. Khan MSR, Kadoya Y (2021) Loneliness during the COVID-19 Pandemic: A Comparison between Older and Younger People. International Journal of Environmental Research and Public Health 18: 7871.

**S2 Table. Detailed descriptive statistics for “actions taken in response to the coronavirus”**

| Item | Lonely | Not lonely | Means comparison |
| --- | --- | --- | --- |
|  | % | % | p value |
| Washed or sanitized hands | 97.49% | 96.55% | 0.078 |
| Kept six feet distance from those outside my household | 95.55% | 94.49% | 0.120 |
| Worn a face mask | 92.62% | 89.26% | <0.001 |
| Avoided public or crowded places | 89.84% | 85.10% | <0.001 |
| Avoided some or all restaurants | 85.73% | 79.80% | <0.001 |
| Canceled or postponed pleasure, social, or recreational activities | 83.79% | 76.32% | <0.001 |
| Avoided contact with high-risk people | 71.40% | 63.40% | <0.001 |
| Prayed | 60.47% | 58.58% | 0.206 |
| Wiped packages entering my home | 53.24% | 49.82% | <0.001 |
| Canceled or postponed dentist or other appointment | 52.12% | 42.89% | <0.001 |
| Canceled a doctor appointment | 50.80% | 41.33% | <0.001 |
| Stockpiled food or water | 38.83% | 28.53% | <0.001 |
| Canceled or postponed work activities | 21.78% | 17.70% | 0.001 |
| Worked from home | 17.47% | 17.58% | 0.924 |
| Canceled outside housekeepers or caregivers | 16.01% | 12.85% | 0.003 |
| Visited a doctor or hospital | 13.01% | 12.25% | 0.452 |
| Stayed home because I felt unwell | 8.63% | 4.59% | <0.001 |
| Studied from home | 7.72% | 7.38% | 0.669 |
| Canceled or postponed school activities | 5.50% | 3.88% | 0.009 |

Note: Question text “Which of the following measures, if any, are you taking in response to the coronavirus?”

## S3 Table. Detailed descriptive statistics for the “personal plans changed”

| Item | Lonely | Not lonely | Means comparison |
| --- | --- | --- | --- |
|  | % | % | p value |
| Domestic travel restrictions or bans | 72.45% | 60.93% | <0.001 |
| International travel restrictions or bans | 44.24% | 32.34% | <0.001 |
| Ban on gatherings of 50 people or more | 34.21% | 28.15% | <0.001 |
| Ban on gatherings of 250 people or more | 33.76% | 29.06% | 0.001 |
| Pre-K or child care closure | 33.09% | 28.57% | 0.001 |
| K-12 school closure | 33.02% | 28.09% | <0.001 |
| Closure of bars | 32.76% | 28.25% | 0.001 |
| College or training closure | 32.64% | 28.90% | 0.008 |
| Other reduced public services | 32.19% | 28.19% | 0.004 |
| Closure of work | 30.76% | 26.50% | 0.002 |
| Closure of other businesses | 30.73% | 26.59% | 0.003 |
| Canceled sport events | 30.71% | 27.48% | 0.020 |
| Reduced public transportation | 30.18% | 27.57% | 0.061 |
| Closure of restaurants | 29.84% | 27.46% | 0.085 |
| Ban on gatherings of 10 people or more | 29.78% | 27.57% | 0.111 |
| Quarantine requirements or stay-at-home orders | 29.64% | 25.51% | 0.002 |
| Closure of place of worship | 29.32% | 27.32% | 0.150 |
| Work from home requirements | 28.89% | 26.66% | 0.102 |
| Closure of gyms or fitness facilities | 28.30% | 27.72% | 0.677 |

Note: Question text: “In the past 7 days, have your personal plans been changed or affected by the following types of restrictions, or not?”

**S4 Table. Dates used to create “Reopened” variable for 10 states**

| State | Reopening date | Source of date |
| --- | --- | --- |
| Reopened by/during wave 1: COVID Impact Survey April 20-26 | | |
| Missouri | April 24 | Missouri Department of Health & Senior Services <https://health.mo.gov/living/healthcondiseases/communicable/novel-coronavirus/> |
| Texas | April 24 | Texas Department of State Health Services <https://dshs.texas.gov/coronavirus/> |
|  | | |
| Reopened by/during wave 2: COVID Impact Survey May 4-17 | | |
| Colorado | April 26 | Colorado Department of Public Health & Environment <https://covid19.colorado.gov/> |
| Montana | April 27 | Montana Department of Public Health & Human Services <https://dphhs.mt.gov/publichealth/cdepi/diseases/coronavirusmt> |
| Florida | May 4 | Florida Health <https://floridahealthcovid19.gov> |
| Louisiana | May 15 | Louisiana Department of Health <https://ldh.la.gov/Coronavirus/> |
| Oregon | May 15 | Oregon Health Authority Public Health Division <https://www.oregon.gov/oha/ph/pages/index.aspx> |
|  | | |
| Reopened by/during wave 3: COVID Impact Survey May 30-June 8, 2020 | | |
| New York | May 27 | New York Department of Health <https://coronavirus.health.ny.gov/home> |
| Minnesota | May 17 | Minnesota Department of Health <https://www.health.state.mn.us/diseases/coronavirus/> |
|  | | |
| Not reopened during the study period | | |
| California | After June 8^th^ | California Department of Public Health <https://www.cdph.ca.gov/Programs/CID/DCDC/Pages/Immunization/ncov2019.aspx> |

Note: COVID Impact Survey dates: April 20-26, May 4-17, May 30-June 8, 2020

**S5 Table. Coefficients of OLS regression of loneliness on liquidity constraints, actions taken, plans changed, and interaction terms, full results**

| Variables | (1)  Loneliness | (2)  Loneliness | (3)  Loneliness | (4)  Loneliness |
| --- | --- | --- | --- | --- |
|  | Coef. (S.E.) | Coef. (S.E.) | Coef. (S.E.) | Coef. (S.E.) |
| Annual household income | -0.004 (0.002) p=0.114 | -0.003 (0.002)  p=0.186 | -0.007*** (0.002), p=0.008 | -0.005 (0.008) p=0.528 |
| Lack emergency savings | 0.130*** (0.023)  p<0.001 | -- | 0.120*** (0.023) p<0.001 | 0.152* (0.077) p=0.050 |
| Individual items: |  |  |  |  |
| Put it on my credit card and pay it off in full at the next statement |  | -0.053* (0.023)  p=0.024 |  |  |
| Use money currently in my checking or savings account or with cash |  | -0.008 (0.021)  p=0.674 |  |  |
| Put it on my credit card and pay it off over time |  | 0.124*** (0.029)  p<0.001 |  |  |
| Use money from a bank loan or line of credit |  | 0.038 (0.061)  p=0.355 |  |  |
| Borrow from a friend or family member |  | -0.060 (0.065)  p=0.355 |  |  |
| Use a payday loan, deposit advance or overdraft |  | 0.078 (0.117)  p=0.502 |  |  |
| Sell something |  | 0.160* (0.072)  p=0.027 |  |  |
| I would not be able to pay for it right now |  | 0.081 (0.045)  p=0.073 |  |  |
| Actions taken |  |  | 0.020*** (0.003) p<0.001 | 0.021* (0.008)  p=0.010 |
| Plans changed |  |  | 0.011*** (0.002) p<0.001 | 0.013** (0.005) p=0.023 |
| Interaction terms: |  |  |  |  |
| Actions taken * Lack emergency saving |  |  |  | -0.003 (0.008) p=0.693 |
| Plans changed * Lack emergency saving |  |  |  | -0.0002 (0.006) p=0.974 |
| Actions taken * Income |  |  |  | 0.000 (0.0009) p=0.994 |
| Plans changed * Income |  |  |  | -0.0003 (0.0006) p=0.627 |
| Health-related controls: |  |  |  |  |
| Respondent had COVID-19 | 0.049 (0.158)  p=0.753 | 0.045 (0.158)  p=0.774 | 0.048 (0.157)  p=0.756 | 0.049 (0.157) p=0.755 |
| Household member had COVID-19 | -0.069 (0.152) p=0.647 | -0.077 (0.152) p=0.608 | -0.093 (0.151) p=0.536 | -0.093 (0.151) p=0.538 |
| Number comorbidities | 0.043*** (0.005) p<0.000 | 0.042*** (0.005) p<0.000 | 0.041*** (0.005) p<0.000 | 0.041*** (0.005) p<0.001 |
| Financial controls: |  |  |  |  |
| Number health insurance | 0.015 (0.009) p=0.110 | 0.015 (0.009) p=0.112 | 0.009 (0.009) p=0.315 | 0.009 (0.009) p=0.313 |
| Number financial assistance | 0.019* (0.008) p=0.028 | 0.018* (0.008) p=0.031 | 0.013* (0.008) p=0.131 | 0.013 (0.008) p=0.132 |
| Socio-demographic controls: |  |  |  |  |
| Age 75 plus | -0.091*** (0.021) p<0.001 | -0.086*** (0.021) p<0.001 | -0.085*** (0.020) p<0.001 | -0.086*** (0.020) p<0.001 |
| Male | -0.149*** (0.019) p<0.001 | -0.149*** (0.019) p<0.001 | -0.127*** (0.019) p<0.001 | -0.127*** (0.019) p<0.001 |
| Non-Hispanic White | Omitted | Omitted | Omitted | Omitted |
| Non-Hispanic Black | -0.235*** (0.038), p<0.001 | -0.239*** (0.039), p<0.001 | -0.252*** (0.038), p<0.001 | -0.232*** (0.038), p<0.001 |
| Non-Hispanic Other | -0.050 (0.046) p=0.277 | -0.050 (0.046) p=0.283 | -0.065 (0.046) p=0.161 | -0.064 (0.046) p=0.166 |
| Hispanic | -0.059 (0.049) p=0.226 | -0.063 (0.049) p=0.195 | -0.081 (0.049) p=0.095 | -0.082 (0.049) p=0.094 |
| No HS diploma | Omitted | Omitted | Omitted | Omitted |
| HS graduate or equivalent | -0.134* (0.066) p=0.044 | -0.133* (0.066) p=0.046 | -0.135* (0.066) p=0.040 | -0.135* (0.066) p=0.042 |
| Some college | -0.125 (0.064) p=0.052 | -0.123 (0.064) p=0.056 | -0.137 (0.063) p=0.031 | -0.137* (0.064) p=0.032 |
| BA or above | -0.099 (0.064) p=0.124 | -0.094 (0.065) p=0.149 | -0.129 (0.064) p=0.045 | -0.129* (0.064) p=0.046 |
| Household size | -0.002 (0.011) p=0.834 | -0.002 (0.011) p=0.829 | -0.007 (0.011) p=0.514 | -0.007 (0.011) p=0.509 |
| Census region |  |  |  |  |
| New England | Omitted | Omitted | Omitted | Omitted |
| Mid-Atlantic | 0.100 (0.092) p=0.280 | 0.102 (0.092) p=0.267 | 0.101 (0.092) p=0.270 | 0.100 (0.092) p=0.273 |
| EN Central | 0.141 (0.091) p=0.124 | 0.145 (0.091) p=0.113 | 0.147 (0.091) p=0.105 | 0.147 (0.091) p=0.106 |
| WN Central | 0.073 (0.093) p=0.436 | 0.076 (0.093) p=0.417 | 0.078 (0.093) p=0.401 | 0.078 (0.093) p=0.402 |
| S Atlantic | 0.101 (0.091) p=0.266 | 0.104 (0.091) p=0.252 | 0.113 (0.090) p=0.211 | 0.113 (0.091) p=0.214 |
| ES Central | 0.067 (0.098) p=0.494 | 0.068 (0.098) p=0.489 | 0.068 (0.097) p=0.482 | 0.068 (0.097) p=0.486 |
| WS Central | 0.075 (0.093) p=0.419 | 0.077 (0.093) p=0.408 | 0.082 (0.093) p=0.375 | 0.082 (0.093) p=0.377 |
| Mountain | 0.091 (0.092) p=0.323 | 0.094 (0.092) p=0.310 | 0.108 (0.092) p=0.242 | 0.107 (0.092) p=0.244 |
| Pacific | 0.153 (0.093) p=0.100 | 0.155 (0.093) p=0.095 | 0.160 (0.092) p=0.083 | 0.160 (0.092) p=0.084 |
| Wave 1 4/20-26/2020 | Omitted | Omitted | Omitted | Omitted |
| Wave 2 5/4-10/2020 | 0.010 (0.022) p=0.652 | 0.008 (0.022) p=0.696 | 0.013 (0.022) p=0.546 | 0.013 (0.022) p=0.555 |
| Wave 3 6/1-8/2020 | -0.019 (0.023) p=0.424 | -0.020 (0.023) p=0.400 | -0.004 (0.023) p=0.851 | -0.004 (0.023) p=0.836 |
| Rural residence | Omitted | Omitted | Omitted | Omitted |
| Suburban residence | 0.033 (0.047) p=0.477 | 0.031 (0.047) p=0.503 | 0.023 (0.047) p=0.623 | 0.022 (0.047) p=0.631 |
| Urban residence | 0.086* (0.043) p=0.048 | 0.087* (0.043) p=0.045 | 0.067 (0.043) p=0.121 | 0.067 (0.043) p=0.123 |
| Survey language is English | -0.076 (0.133) p=0.566 | -0.066 (0.133) p=0.620 | -0.044 (0.132) p=0.735 | -0.046 (0.133) p=0.727 |
| Survey mode is web | 0.030 (0.029), p=0.289 | 0.033 (0.029), p=0.257 | 0.032 (0.029), p=0.262 | 0.031 (0.029), p=0.275 |
| Constant | 1.299*** (0.176) p<0.001 | 1.320*** (0.178) p<0.001 | 1.090*** (0.178) p<0.001 | 1.073*** (0.187) p<0.001 |
| F test (df) | 9.50*** (30) | 8.18*** (37) | 11.15*** (32) | 9.92*** (36) |
| Adj. R-squared | 0.048 | 0.044 | 0.054 | 0.053 |
| Observations | 5,664 | 5,664 | 5,664 | 5,664 |

Note: * p<0.05, ** p<0.01, *** p<0.001

**S6 Table. Coefficients of OLS regression of loneliness on liquidity constraints, actions taken and plans changed, and interaction terms: categorical coding, continuous low and high bracket coding of the income variable**

| Variables | (1)  Loneliness | (3)  Loneliness  (Income is coded continuous, at the low bracket) | (4)  Loneliness  (Income is coded continuous, at the high bracket) |
| --- | --- | --- | --- |
|  | Coef. (S.E.) | Coef. (S.E.) | Coef. (S.E.) |
| Annual household income (cont.) |  | -0.004 (0.002) p=0.137 | -0.003 (0.002) p=0.107 |
| Under $10,000 | omitted |  |  |
| $10,000 to under $20,000 | -0.072 (0.069) p=0.300 |  |  |
| $20,000 to under $30,000 | -0.051 (0.067) p=0.444 |  |  |
| $30,000 to under $40,000 | -0.069 (0.068) p=0.311 |  |  |
| $40,000 to under $50,000 | -0.066 (0.070) p=0.346 |  |  |
| $50,000 to under $75,000 | -0.077 (0.067) p=0.249 |  |  |
| $75,000 to under $100,000 | -0.117 (0.069) p=0.089 |  |  |
| $100,000 to under $150,000 | -0.101 (0.070) p=0.149 |  |  |
| $150,000 or more | -0.103 (0.072) p=0.156 |  |  |
| Lack emergency savings | 0.128*** (0.024)  p<0.001 | 0.131*** (0.023) p<0.001 | 0.129*** (0.023) p<0.001 |
| Control measures: |  |  |  |
| Health-related measures | Yes | Yes | Yes |
| Financial, socio-demographic measures | Yes | Yes | Yes |
| F test (df) | 7.77*** (37) | 9.49*** (30) | 9.51*** (30) |
| Adj. R-squared | 0.042 | 0.048 | 0.043 |
| Observations | 5,664 | 5,664 | 5,664 |

Note: * p<0.05, ** p<0.01, *** p<0.001

Health-related controls: Respondent had COVID-19; Household member had COVID-19; Number comorbidities

Financial, socio-demographic controls: Number health insurance; Number financial assistance; Age 75 plus; Gender; Race/Ethnicity; Educational attainment; Household size; Census region; Wave; Urban-rural residence; Survey language; Survey mode

Continuous coding, lower bounds of the income brackets: $0, $10,000, $20,000, $30,000, $40,000, $50,000, $75,000, $100,000, $150,000

Continuous coding, the upper bounds of the income brackets: $10,000, $20,000, $30,000, $40,000, $50,000, $75,000, $100,000, $150,000 (twice)

**S7 Table. Coefficients of OLS regression of loneliness on liquidity constraints, actions taken, plans changed, and interaction terms, alternative coding of loneliness outcome measure**

| Variables | (1)  Loneliness,  focal results, OLS | (2)  Loneliness,  Upper-level coding, OLS | (3)  Loneliness,  Binary coding, binary logistic regression |
| --- | --- | --- | --- |
|  | Coef. (S.E.) | Coef. (S.E.) | OR (S.E.) |
| Annual household income | -0.005 (0.008) p=0.528 | -0.007 (0.014) p=0.572 | 0.975 (0.030)  p=0.436 |
| Lack emergency savings | 0.152* (0.077) p=0.050 | 0.277* (0.131) p=0.035 | 1.584 (0.438)  p=0.096 |
| Actions taken | 0.021* (0.008)  p=0.010 | 0.028* (0.013)  p=0.040 | 1.092** (0.031) p=0.002 |
| Plans changed | 0.013** (0.005) p=0.023 | 0.018 (0.010) p=0.065 | 1.054** (0.021) p=0.009 |
| Interaction terms: |  |  |  |
| Actions taken * Lack emergency saving | -0.003 (0.008) p=0.693 | -0.007 (0.014) p=0.629 | 0.980 (0.029)  p=0.509 |
| Plans changed * Lack emergency saving | -0.0002 (0.006) p=0.974 | -0.00004 (0.010) p=0.996 | 0.996 (0.019)  p=0.868 |
| Actions taken * Income | 0.000 (0.0009) p=0.994 | 0.00002 (0.001) p=0.988 | 1.001 (0.003)  p=0.868 |
| Plans changed * Income | -0.0003 (0.0006) p=0.627 | -0.0003 (0.001) p=0.773 | 0.998 (0.002)  p=0.495 |
| Health-related controls: | Yes | Yes | Yes |
| Financial controls: | Yes | Yes | Yes |
| F test/Chi2 (df=36) | 9.92*** | 8.02*** | 361.86*** |
| Adj./Pseudo R-squared | 0.053 | 0.042 | 0.056 |
| Observations | 5,664 | 5,664 | 5,664 |

Note: * p<0.05, ** p<0.01, *** p<0.001

**S8 Table. Coefficients of OLS regression of loneliness on liquidity constraints, actions taken, plans changed, and interaction terms: Subsample regressions**

| Variables | Full results  Loneliness | Female subsample | Younger, age 65-74 subsample | Non-white subsample |
| --- | --- | --- | --- | --- |
|  | Coef. (S.E.) | Coef. (S.E.) | Coef. (S.E.) | Coef. (S.E.) |
| Annual household income | -0.005 (0.008) p=0.528 | -0.004 (0.014) p=0.748 | -0.012 (0.010) p=0.232 | -0.00006 (0.022) p=0.998 |
| Lack emergency savings | 0.152* (0.077) p=0.050 | 0.240* (0.121) p=0.049 | 0.151 (0.095) p=0.111 | 0.147 (0.173) p=0.395 |
| Actions taken | 0.021* (0.008)  p=0.010 | 0.029* (0.013)  p=0.023 | 0.015 (0.010)  p=0.141 | 0.028 (0.019)  p=0.158 |
| Plans changed | 0.013** (0.005) p=0.023 | 0.017 (0.009) p=0.056 | 0.018* (0.007) p=0.016 | 0.010 (0.012) p=0.408 |
| Interaction terms: |  |  |  |  |
| Actions taken * Income | 0.0000006 (0.0009) p=0.994 | -0.00006 (0.001) p=0.966 | 0.0007 (0.001) p=0.486 | -0.001 (0.002) p=0.544 |
| Plans changed * Income | -0.0003 (0.0006) p=0.627 | -0.0002 (0.001) p=0.798 | -0.0003 (0.0007) p=0.650 | -0.0005 (0.001) p=0.675 |
| Actions taken * Lack emergency savings | -0.003 (0.008) p=0.693 | -0.007 (0.013) p=0.595 | -0.001 (0.010) p=0.874 | -0.006 (0.018) p=0.727 |
| Plans changed * Lack emergency savings | -0.0002 (0.006) p=0.974 | -0.008 (0.008) p=0.354 | -0.004 (0.007) p=0.548 | -0.008 (0.011) p=0.448 |
| Control measures: |  |  |  |  |
| Health-related measures | Yes | Yes | Yes | Yes |
| Financial, socio-demographic measures | Yes | Yes | Yes | Yes |
| F test (df) | 9.92*** (36) | 5.26*** (35) | 8.03*** (35) | 2.10** (35) |
| Adj. R-squared | 0.053 | 0.047 | 0.058 | 0.040 |
| Observations | 5,664 | 2,966 | 3,985 | 904 |

Note: * p<0.05, ** p<0.01, *** p<0.001

Health-related controls: Respondent had COVID-19; Household member had COVID-19; Number comorbidities

Financial, socio-demographic controls: Number health insurance; Number financial assistance; Age 75 plus; Gender; Race/Ethnicity; Educational attainment; Household size; Census region; Wave; Urban-rural residence; Survey language; Survey mode
